# Circulating concentrations of glycocalyx degradation products in preeclampsia

**DOI:** 10.3389/fphys.2022.1022770

**Published:** 2022-10-13

**Authors:** Niclas Carlberg, Catherine Cluver, Camilla Hesse, Sven-Egron Thörn, Robin Gandley, Tor Damén, Lina Bergman

**Affiliations:** ^1^ Department of Anesthesiology and Intensive Care Medicine, Sahlgrenska University Hospital Östra, Gothenburg, Sweden; ^2^ Department of Obstetrics and Gynecology, Institute of Clinical Sciences, Sahlgrenska Academy, University of Gothenburg, Gothenburg, Sweden; ^3^ Department of Obstetrics and Gynecology, Stellenbosch University, Cape Town, South Africa; ^4^ Translational Obstetrics Group, Department of Obstetrics and Gynaecology, University of Melbourne, Melbourne, VI, Australia; ^5^ Mercy Perinatal, Mercy Hospital for Women, Heidelberg, VI, Australia; ^6^ Department of Laboratory Medicine, Institute of Biomedicine, Gothenburg University, Gothenburg, Sweden; ^7^ Department of Anesthesiology and Intensive Care Medicine, Institute of Clinical Sciences at the Sahlgrenska Academy, University of Gothenburg, Gothenburg, Sweden; ^8^ Department of Obstetrics, Gynecology and Reproductive Sciences and Magee Womens Research Institute, University of Pittsburgh, Pittsburgh, PA, United States; ^9^ Section of Cardiothoracic Anesthesia and Intensive Care, Sahlgrenska University Hospital, Gothenburg, Sweden; ^10^ Department of Women’s and Children’s Health, Uppsala University, Uppsala, Sweden

**Keywords:** preeclampsia, glycocalyx, syndecan 1, hyaluronic acid, thrombomodulin, hypertension in pregnancy, endothelial dysfuction

## Abstract

**Background and Objectives:** Preeclampsia is a multisystem disorder that affects maternal endothelium. The glycocalyx lines and protects the endothelial surface. In severe systemic diseases, like sepsis, it is shed and glycocalyx degradation products can be detected in increased concentrations in plasma. The aim of this study was to compare circulating concentrations of glycocalyx degradation products in degrees of preeclampsia severity.

**Study design**: In this observational study, we included women from the South African PROVE biobank. Women were divided into normotensive controls, women with preeclampsia without end-organ complications, women with a single end-organ complication and women with multiple end-organ complications. Plasma samples taken at inclusion after diagnosis (preeclampsia cases) or at admission for delivery (normotensive controls) were analyzed with ELISA for syndecan-1, hyaluronic acid and thrombomodulin and compared between groups.

**Results:** Women with preeclampsia (*n* = 47) had increased plasma concentrations of hyaluronic acid (100.3 ng/ml IQR 54.2–204 vs*.* 27.0 ng/ml IQR (13.5–66.6), *p* < 0,001) and thrombomodulin (4.22 ng/ml IQR 3.55–5.17 vs*.* 3.49 ng/ml IQR 3.01–3.68, *p* = 0.007) but not syndecan-1 compared with normotensive women (*n* = 10). There were no differences in plasma concentration in any of these biomarkers in women with preeclampsia with no end-organ complications (*n* = 10) compared with women with preeclampsia and one end-organ complication (*n* = 24). Women with preeclampsia with two or more end-organ complications (*n* = 13) had increased plasma concentrations of thrombomodulin (5.46 ng/ml, IQR 4.85–7.83 vs*.* 4.66 ng/ml, IQR 3.45–4.88, *p* = 0.042) compared with women with preeclampsia and no end-organ complications.

**Conclusion:** Thrombomodulin was associated with disease severity and may be valuable for risk-stratifying women with preeclampsia.

## Introduction

Preeclampsia is a pregnancy-specific disorder that leads to the development of hypertension and multi-organ injury after 20 weeks of gestation (Chappell et al., 2021). It complicates about 5% of all pregnancies and is associated with 10–15% of all direct maternal deaths (Duley, 2009; Chappell et al., 2021). In low-middle-income countries, 25% of stillbirths and neonatal deaths are caused by preeclampsia (Duley, 2009). The pathophysiology is not fully elucidated but most agree that variable degrees of placental malperfusion result in the release of soluble factors into the maternal circulation, which results in maternal endothelial dysfunction (Tomimatsu et al., 2019).

Normal endothelium is covered by a thin gel-like layer called the glycocalyx, which is important in regulating capillary wall permeability, vessel tone, coagulation and inflammation (Ushiyama et al., 2016). In pathologies such as sepsis or bleeding, endothelial cell homeostasis is disturbed and glycocalyx is shed. Glycocalyx degradation products include syndecan-1, hyaluronic acid and thrombomodulin (Ushiyama et al., 2016). Shedding causes capillary leakage resulting in edema and proteinuria, dysregulation of vessel tone leading to hypertension and impaired microcirculation, activation of the coagulation system causing consumption of platelets, and inflammatory changes (Becker et al., 2015; Anand et al., 2016; Ushiyama et al., 2016; Hippensteel et al., 2019).

Syndecan-1 is expressed by endothelial cells and by the syncytotrophoblast cells of the placenta (Hofmann-Kiefer et al., 2013a; Heyer-Chauhan et al., 2014). Syndecan-1 concentrations in maternal plasma increase during pregnancy and at term reach concentrations comparable to those found in sepsis (Gandley et al., 2016; Kuessel et al., 2019). Before the onset of preeclampsia, some studies have shown decreased plasma concentrations of syndecan-1 compared to normotensive pregnancies, while others have not (Gandley et al., 2016; Kuessel et al., 2019; Greeley et al., 2020). In women with preeclampsia circulating concentrations of syndecan-1 have been shown to be similar, decreased or increased compared with normotensive controls (Hofmann-Kiefer et al., 2013b; Gandley et al., 2016; Kornacki et al., 2019; Weissgerber et al., 2019; Hassani Lahsinoui et al., 2021).

Hyaluronic acid is a glycosaminoglycan and an important component of the endothelial glycocalyx (Alberts, 1989). Serum concentrations increase slightly during uncomplicated pregnancy and labor (Kobayashi et al., 1999). Circulating concentrations of hylauronic acid are increased in preeclampsia compared to normotensive pregnancies (Osmers et al., 1998; Berg et al., 2001; Hofmann-Kiefer et al., 2013b; Kornacki et al., 2019; Weissgerber et al., 2019).

Thrombomodulin is a cofactor for thrombin when activating protein C (Levi and Van Der Poll, 2013). Plasma concentrations have been shown to be increased in preeclampsia of different severity, when compared to normotensive pregnancies (Hsu et al., 1993; Minakami et al., 1993; Bontis et al., 1995; Shaarawy and Didy, 1996; Rousseau et al., 2009).

No studies have assessed whether the severity of preeclampsia, which may be proportional to the degree of endothelial dysfunction, can be reflected by circulating concentrations of endothelial glycocalyx degradation products. The aim of this study was to investigate plasma concentrations of syndecan-1, hyaluronic acid and thrombomodulin in preeclampsia of different severity.

## Materials and methods

### Study cohort

**FIGURE 1:**
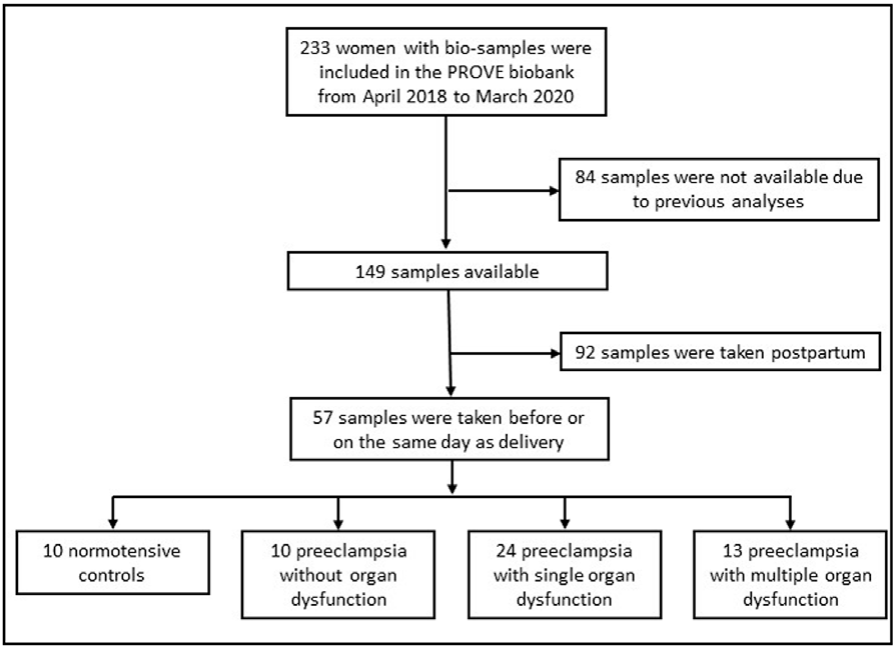
Flowchart of the population.

A flow chart of the study population is shown in Figure 1. Women with singleton pregnancies during 2018–2020 who were recruited to the Preeclampsia Obstetric Adverse Events (PROVE) biobank at Tygerberg Hospital, Cape Town, South Africa before delivery were included (Bergman et al., 2021). Exclusion critera included known neurological or cardiac disease. For normotensive women, additional exclusion criteria included diabetes mellitus and chronic hypertension. Preeclampsia was defined according to the American College of Obstetricians and Gynecologists Practice Bulletin but significant proteinuria (protein creatinine ratio ≥ 30 mg/mmol (0.3 mg/mg) or ≥ 0.3 g protein in a 24 h urine collection or urine dipstick >2 + on more than one occasion) was also required for diagnosis (Gestational Hypertension and Preeclampsia, 2020). Eclampsia, neurological deficits, pulmonary edema, renal impairment, hemolysis and elevated liver enzymes and low platelet syndrome (HELLP), elevated liver enzymes, liver rupture and platelets below 100 × 10^9^ were considered as end-organ complications. Eclampsia was defined as generalized tonic clonic seizures in a woman diagnosed with preeclampsia in the absence of another etiology. Multiple neurological complications were assessed as one end-organ complication. HELLP was defined as a platelet count less than 100 × 10^9^/L, aspartate aminotransferase (AST) greater than 70 U/L and lactate dehydrogenase (LD) > 600 U/L or hemolysis on a peripheral blood smear. Pulmonary edema was diagnosed when there was worsening dyspnea, bilateral fine inspiratory crackles on auscultation and features of pulmonary edema on chest x-ray. Serum creatinine above 120 μmol/L was considered as renal impairment. Severe hypertension, defined as a systolic blood pressure ≥ 160 mm Hg systolic and/or a diastolic blood pressure ≥ 110 mm Hg, was not considered an end-organ complication. Blood pressure and AST were recorded as highest values and platelets and hemoglobin (Hb) as lowest values, retrieved from the medical charts before delivery.

Women with preeclampsia were divided into three groups: preeclampsia without end-organ complications, preeclampsia with a one end-organ complication and preeclampsia with two or more end-organ complications. After inclusion and blood-sampling, any later complications were recorded but women remained in their initial group. No women with normotensive pregnancies developed hypertension. Women were followed up from recruitment until discharge after delivery.

Baseline data were obtained by interview and extraction from medical records. All data were entered and stored on a Research Electronic Data Capture database (Harris et al., 2019) and double checked for accuracy.

### Sample collection

Plasma samples were collected in ethylenediaminetetraacetic acid (EDTA) tubes at inclusion after a diagnosis of preeclampsia or after admission for delivery (normotensive controls). Women were included in the PROVE biobank before or shortly after delivery, but only women with blood samples obtained for PROVE before delivery were included in this study. Samples were centrifuged, aliquoted and frozen at -80°C and were shipped frozen to the University of Gothenburg for analysis.

### Biomarker assays

Plasma concentrations of syndecan-1, hyaluronic acid and thrombomodulin were measured using commercial ELISA-kits following the manufacturer’s instructions. Syndecan-1 was analyzed with kits from Diaclone (Human CD138 ELISA kit, catalogue number 851.620.001). Hyaluronic acid and thrombomodulin were analyzed with kits from R&D-systems (hyaluronic acid: Hyaluronan Quantikine ELISA kit catalogue number DHYAL0 and thrombomodulin: Human Thrombomodulin/BDCA-3 Quantikine ELISA Kit catalogue number DTHBD0). Calibrators and samples were run in duplicates. A quality control (QC) sample was run in duplicates on every plate. For syndecan-1, 19 samples were undiluted, 30 diluted 1:12, and eight were diluted 1:32. For analyses of hylaruronic acid and thrombomodulin samples were diluted 1:10. Absorbance was read with a Multiscan GO instrument, using SkanIT software 4.1. All data were double checked for accuracy.

### Statistical methods

Sample size calculation was based on data from Berg, Hoffman and Weissberger, (Berg et al., 2001; Hofmann-Kiefer et al., 2013b; Weissgerber et al., 2019) assuming a log-normal distribution of the response variables. This resulted in an estimated sample size of 22 women (*n* = 11 per group) to achieve 80% power with a significance level of 0.05 to detect a significant difference in the distribution of hyaluronic acid between women with preeclampsia and normotensive controls.

Background data and outcomes are presented as medians with interquartile range or numbers with percentages according to distribution of the variable. Differences between groups were analyzed by Kruskal–Wallis as a global test and Mann-Whitney U-test for pairwise comparisons with Bonferroni correction. For syndecan-1, differences between groups were adjusted for gestational age at sampling by a robust multiway ANOVA (parameter estimates with robust standard error to adjust for differences in variance) which was conducted with gestational age as continuous covariate. Hyaluronic acid and thrombomodulin are not known to vary with gestational age.

In all hypothesis tests, a two-sided *p*-value of less than 0.05 was considered statistically significant. Data and statistical analyses were performed using SPSS (IBM SPSS statistics version 28.0.1.0 (142), Chicago, Illinois, United States).

### Ethics approval and registration details

Approval was obtained from the Health Research and Ethics Committee at Stellenbosch University (PROVE Biobank protocol number N18/03/034, substudy protocol number N22/02/01, Federal Wide assurance number 00001372, Institutional Review Board number IRB0005239). All participants or their guardians signed informed consent. The biobank is registered (ISRCTN10623443) and the protocol is published (Bergman et al., 2021).

## Results

### Background characteristics

Maternal characteristics and pregnancy outcomes are presented in Table 1. In women with multiple end-organ dysfunction, there was one maternal death, 6/13 (46%) of neonates were stillborn and HELLP syndrome occurred in 10/13 (77%). Eclampsia occurred in 20/24 (83%) and 9/13 (69%) of women with one and multiple end-organ complications respectively. Intubation was required in 3/24 (13%) and 3/13 (23%) in women with a one and multiple end-organ complications respectively. All women who required intubation had Glascow Coma Scale < 13 and additional neurological complications.

**TABLE 1:** Background characteristics of the population.

	Normotensive control	Preeclampsia without end-organ complications	Preeclampsia and a single end-organ complication	Preeclampsia and multiple end-organ complications
*n*	10	10	24	13
Age	30 (25–33)	23 (21–32)	18 (17–24)	25 (21–28)
Nulliparous	4 (40%)	6 (60%)	19 (79%)	7 (54%)
BMI (kg/m^2^)	25 (21–31)	28 (24–39)	25 (22–27)	27 (25–30)
Diabetes	0 (0%)	0 (0%)	1 (4%)	0 (0%)
Chronic hypertension	0 (0%)	3 (30%)	2 (8%)	3 (23%)
Renal disease	0 (0%)	0 (0%)	1 (4%)	0 (0%)
Smoking	3 (30%)	1 (10%)	5 (21%)	1 (8%)
Alcohol use	2 (20%)	0 (0%)	5 (21%)	0 (0%)
Methamphetamine	0 (0%)	0 (0%)	0 (0%)	1 (8%)
HIV	3 (30%)	2 (20%)	3 (13%)	4 (31%)
SBP (mmHg)	112 (109–124)	168 (156–175)	166 (146–184)	168 (163–190)
DBP (mmHg)	64 (53–76)	98 (88–117)	100 (94–114)	105 (101–120)
Laboratory tests				
Platelets (x 10^9^/L)	337 (218–373)	198 (164–270)	201 (176–227)	75 (48–155)
AST (IU)	*n* = 0	25 (23–26)	26 (22–46)	122 (40–222)
Hemoglobin g/L	9.4 (8.4–10.6)	11.8 (11.0–12.5)	11.6 (9.8–13.5)	11.3 (8.8–14.0)
GA at delivery	37 + 6 (34+3–39 + 2)	33 + 5 (30+5–38 + 1)	34 + 2 (31+6–37 + 0)	30 + 3 (27+1–33 + 0)
Mode of delivery				
Vaginal delivery	2 (20%)	3 (30%)	6 (25%)	6 (46%)
Caesarean section	8 (80%)	7 (70%)	18 (75%)	7 (54%)
Liveborn	10 (100%)	7 (70%)	22 (92%)	7 (54%)
Birth weight (g)	2915 (2070–3411)	1825 (838–3234)	2185 (1456–3008)	1210 (898–1732)
Maternal complications				
Maternal death	0 (0%)	0 (0%)	0 (0%)	1 (8%)
Severe hypertension	0 (0%)	4 (40%)	4 (17%)	9 (69%)
Eclampsia	0 (0%)	0 (0%)	20 (83%)	9 (69%)
Stroke	0 (0%)	0 (0%)	1 (4%)	0 (0%)
GCS < 13	0 (0%)	0 (0%)	4 (17%)	3 (23%)
Cortical blindness	0 (0%)	0 (0%)	1 (4%)	0 (0%)
Pulmonary edema	0 (0%)	0 (0%)	4 (17%)	2 (15%)
HELLP	0 (0%)	1 (10%)	0 (0%)	10 (77%)
Renal impairment	0 (0%)	0 (0%)	0 (0%)	5 (38%)
Dialysis	0 (0%)	0 (0%)	0 (0%)	0 (0%)
Postpartum hemorrhage	1 (10%)	0 (0%)	2 (8%)	2 (15%)
Sepsis	0 (0%)	0 (0%)	2 (8%)	1 (8%)
Intubation	0 (0%)	0 (0%)	3 (13%)	3 (23%)
Venous thromboembolism	0 (0%)	0 (0%)	0 (0%)	1 (8%)
Placental abruption	0 (0%)	0 (0%)	0 (0%)	2 (15%)

Data are presented as count (%) or median (IQR).

AST: aspartate aminotransferase, BMI: body mass index, DBP: diastolic blood pressure, GA: gestational age, GCS: glscow coma scale, HELLP: hemolysis, Elevated liver enzymes, Low platlets, HIV: human immunodeficiency virus, SBP: systolic blood pressure.

### Circulating glycocalyx degradation products

There was no difference in plasma concentrations of syndecan-1 between groups, also after adjustment for gestational age. Women with preeclampsia (*n* = 47) demonstrated a three-fold increase in plasma concentrations of hyaluronic acid (100.3 ng/ml, IQR 54.2–204 vs*.* 27.0 ng/ml, IQR 13.5–66.6 *p* = 0.017) and also increased concentrations of thrombomodulin (4.22 ng/ml, IQR 3.55–5.17 vs*.* 3.49 ng/ml IQR 3.01–3.68 *p* = 0.007) compared with normotensive women (*n* = 10). See Table 2.

**TABLE 2:** Results: Normotensive vs. Preeclampsia.

Biomarker	Unadjusted analyses			Adjusted analysis		
	Normotensive (*n* =10)	Preeclampsia (*n* = 47)	*p*-value	Normotensive (*n* = 10)	Preeclampsia (*n* =47)	*p*-value
Syndecan-1 (ng/ml)	381 (127–541)	461 (321–750)	0.12	5.93 (4.84–6.29)	6.13 (5.77–6.62)	0.11
Hyaluronic acid (ng/ml)	27.0 (13.5–66.6)	100.3 (54.2–204)	<0.001			
Thrombomodulin (ng/ml)	3.49 (3.01–3.68)	4.22 (3.55–5.17)	0.007			

Data are presented as medians with interquartile range. Values for syndecan-1, are presented both as absolute and *ln(syndecan-1)* adjusted for gestational age at blood sampling. Unadjusted analyses are performed by two-tailed Mann Whitney U-test. Adjusted analysis are performed by robust multiway ANOVA.

**FIGURE 2:**
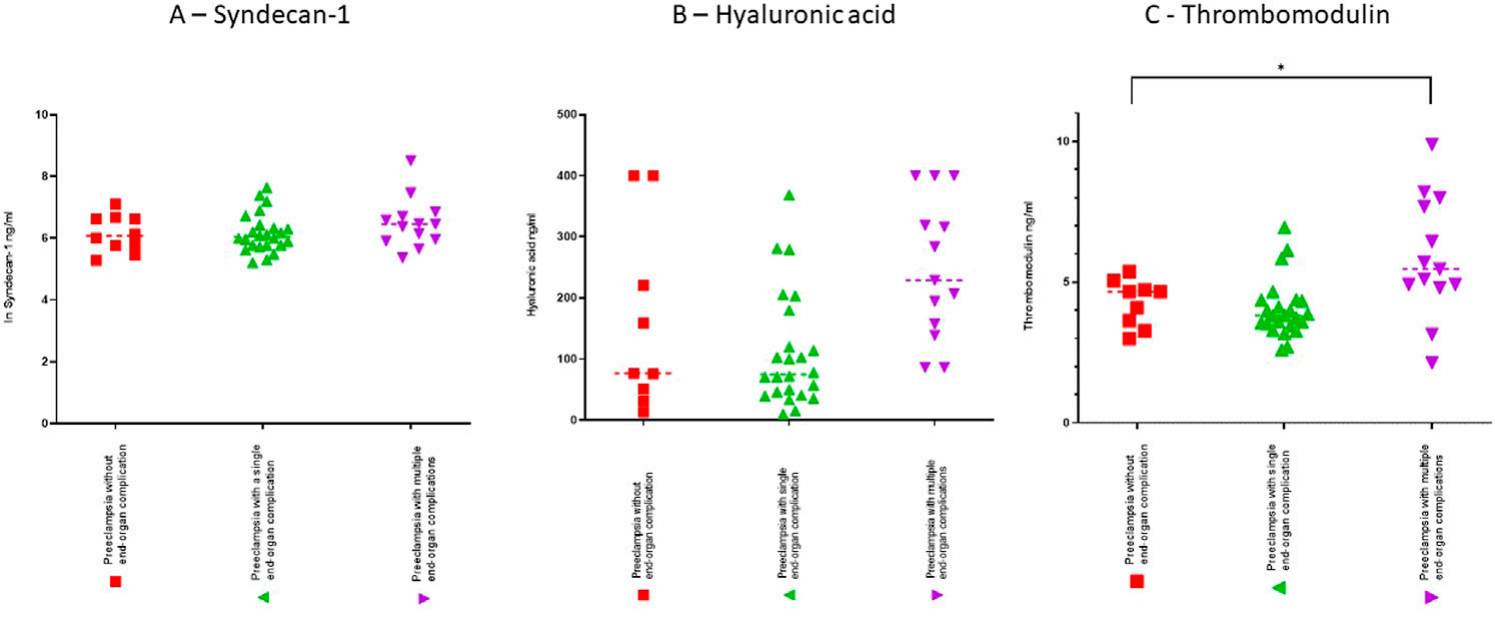
Scatterdots showing plasma concentrations with medians for syndecan-1 **(A)**, hyaluronic acid **(B)** and thrombomodulin **(C)**. Syndecan-1 is presented in a logarithmic scale. Preeclampsia without end-organ complications (*n* = 10), preeclampsia with a single end-organ complication (*n* = 24) and preeclampsia with multiple end-organ complications (*n* = 13). *p < 0.05 vs*.* preeclampsia without end-organ complications.

Differences in biomarkers within the preeclampsia group are presented in Table 3 and Figure 2. There were no differences in plasma concentrations in any of the biomarkers in women with preeclampsia with no end-organ complication (*n* = 10) compared with women with preeclampsia and one end-organ complication (*n* = 24). When comparing women with multiple end-organ complications with women with no end-organ complication, there were no differences in plasma concentrations of syndecan-1 between groups in unadjusted analyses (635 ng/ml, IQR 376–881 vs*.* 433 ng/ml IQR 296–760, *p* = 0.770) or adjusted for gestational age (in logarithmic scale) (6.45, IQR 5.93–6.78 vs. 6.07, IQR 5.68–6.63, *p* = 0.209). Women with preeclampsia and multiple end-organ complications (*n* = 13) had no difference in concentration of hyaluronic acid (229 ng/ml IQR 148–360 vs*.* 76.6 ng/ml IQR 41.1–310 *p* = 0.17) but increased plasma concentrations of thrombomodulin (5.46 ng/ml, IQR 4.85–7.83 vs*.* 4.66 ng/ml, IQR 3.45–4.88, *p* = 0.042) compared with women with preeclampsia and no end-organ complications (*n* = 10).

**TABLE 3:** Results: Preeclampsia of different severity.

Biomarker	Unadjusted				Adjusted			
	Without end-organ complication (*n* = 10)	A single end-organ complication (*n* = 24)	Multiple end-organ complications (*n* = 13)	*p*-value	Without end-organ complication (*n* = 10)	A single end-organ complication (*n* = 24)	Multiple end-organ complications (*n* = 13)	*p*-value
Syndecan-1 (ng/ml)	433 (296–760)	420 (321–602)	635 (376–881)	0.40	6.07 (5.68–6.63)	6.04 (5.78–6.40)	6.45 (5.93–6.78)	0.09
Hyaluronic acid (ng/ml)	76.6 (41.1–310)	75.1 (42.2–165)	229 (148–360)	0.004				
Thrombomodulin (ng/ml)	4.66 (3.45–4.88)	3.81 (3.47–4.36)	5.46 (4.85–7.83)*	0.007				

Data presented as medians with interquartile range. Kruskall Wallis test for global differences between groups with post hoc two-tailed Mann-Whitney U-test and Bonferroni correction for pairwise comparisons. Robust multiway ANOVA, for adjusted analysis. Values for syndecan-1, are presented both as absolute and *ln(syndecan-1)* adjusted for gestational age at blood sampling.

*p < 0.05 vs*.* preeclampsia without end-organ complications.

## Discussion

### Principal findings

Circulating concentrations of thrombomodulin were increased in women with preeclampsia with two or more end-organ complications compared with women with preeclampsia without end-organ complications.

### Results in context

The data on circulating concentrations of syndecan-1 in preeclampsia are conflicting. Syndecan-1 is produced in vast amounts from the placenta (Hofmann-Kiefer et al., 2013a; Gandley et al., 2016; Kuessel et al., 2019). Hofmann-Keifer et al. showed circulating concentrations of syndecan-1 were elevated 159-fold at term in healthy pregnancies compared to age-matched non-pregnant women (Hofmann-Kiefer et al., 2013b). They also showed that women with HELLP-syndrome had plasma concentrations of syndecan-1 that were doubled compared with healthy, term pregnancy, possibly reflecting an extensive endothelial injury in HELLP syndrome (Hofmann-Kiefer et al., 2013b). Interestingly, two studies found lower plasma concentrations in women with early onset preeclampsia compared to normotensive pregnancies and another study did not find any difference (Kornacki et al., 2019; Kuessel et al., 2019; Weissgerber et al., 2019). In a group of women with preeclampsia of unspecified severity, there was no difference in circulating concentrations of syndecan-1 vs*.* normotensive controls (Hassani Lahsinoui et al., 2021). We have no differences between preeclampsia and normotensive pregnancies or within the group of women with preeclampsia. Extra-endothelial origin such as the placenta may be overshadowing the contribution of syndecan-1 concentrations in plasma from an injured endothelium in preeclampsia.

Plasma concentrations of hyaluronic acid have been shown to be increased in both early onset preeclampsia, late onset preeclampsia, severe preeclampsia, eclampsia, and HELLP-syndrome compared with normotensive controls (Osmers et al., 1998; Berg et al., 2001; Kornacki et al., 2019; Weissgerber et al., 2019). None of these studies have compared circulating concentrations of hyaluronic acid within preeclampsia of different severity. In our study, plasma concentrations of hyaluronic acid were increased three-fold in women with preeclampsia compared with normotensive controls. Though, despite higher median values of hyaluronic acid in women with preeclampsia and multiple end-organ complications compared with women with preeclampsia with no end-organ complication, we could not demonstrate a significant difference between the groups. The lack of statistical significance may be due to small groups, but the broad overlapping confidence intervals imply a large variance within groups, potentially precluding hyaluronic acid from acting as a biomarker for disease severity in preeclampsia.

Plasma concentrations of thrombomodulin have been shown to be increased in gestational weeks 24 and 32 before onset of disease in women that later developed preeclampsia compared to women with normotensive pregnancies (Boffa et al., 1998). Seven studies have shown increased circulating concentrations of thrombomodulin in women with preeclampsia compared with normotensive controls (Hsu et al., 1993; Minakami et al., 1993; Bontis et al., 1995; Hsu et al., 1995; Shaarawy and Didy, 1996; Rousseau et al., 2009; Dusse et al., 2013). This was true for both severe preeclampsia and preeclampsia without severe features except for two studies where no differences were found between preeclampsia without severe features and normotensive pregnancies (Hsu et al., 1993; Dusse et al., 2013). One study failed to show any difference between late onset preeclampsia vs*.* normotensive pregnancies, but showed increased circulating concentrations among women with early onset preeclampsia compared to late onset preeclampsia (Alpoim et al., 2018). One study investigated differences in thrombomodulin concentrations in preeclampsia with severe features (definition of severe features not further described) versus preeclampsia without severe features and did not detect any differences in plasma concentrations of thrombomodulin between groups (Boffa et al., 1998). Our research adds to current knowledge by demonstrating that plasma concentrations of thrombomodulin increase by number of end-organ complications in preeclampsia when compared to women with preeclampsia without end-organ complications. This identifies thrombomodulin as a potential candidate to reflect disease severity.

### Research implications

Evidence of endothelial glycocalyx as an important pathophysiological pathway in preeclampsia needs further investigation. Endothelial injury is a dynamic process and time needs to be considered as an important variable when interpreting results of circulating concentrations of glycocalyx degradation products. One measurement of syndecan-1 or hyaluronic acid may not be of value for identifying or predicting worsening of disease, but perhaps the change over time in plasma concentrations could provide important information. Difference over time between two samples taken hours or days apart, may predict disease progress and guide clinical management. Further research is needed. Thrombomodulin may be of value to discern severity of disease by a single test. Future prospective studies are necessary to conclude if thrombomodulin is a valuable biomarker for progression of disease in women with preeclampsia. The physiological importance of altered circulating concentrations of glycocalyx degradation products in the underlying pathophysiology of the endothelial injury in preeclampsia are prospects for future studies.

### Strengths and limitations

Strengths of this study include the unique cohort of women with severe complications of preeclampsia and the robust laboratory method with samples run in duplicates and generally demonstrating good correlations values. Limitation include that the study population includes unproportionally many women with eclampsia, as these women were actively included in the Prove Biobank. The possibility that endothelial glycocalyx shedding is missed or overestimated by increased or decreased hepatic or renal clearance of fragments can not be excluded by the present study design. This is true also for the possibility of glycocalyx degradation products found in plasma originating from other sources than the endothelium.

## Conclusion

Circulating concentrations of glycocalyx degradations products are increased in preeclampsia compared with normotensive pregnancies. Thrombomodulin as a marker of endothelial injury in preeclampsia was associated with severity of disease and may be valuable in risk-stratifying women with preeclampsia.

## Data Availability

The datasets presented in this article are not readily available because access to data needs to be approved by the health ethics committee at Stellenbosch University and in addition, a transfer of data agreement (DTA) is required. Requests to access the datasets should be directed to lina.bergman.2@gu.se.
